# Transcuticular calcium imaging as a tool for the functional study of insect odorant receptors

**DOI:** 10.3389/fnmol.2023.1182361

**Published:** 2023-08-14

**Authors:** Julia Mariette, Amélie Noël, Thierry Louis, Nicolas Montagné, Thomas Chertemps, Emmanuelle Jacquin-Joly, Frédéric Marion-Poll, Jean-Christophe Sandoz

**Affiliations:** ^1^Evolution, Genomes, Behaviour and Ecology, IDEEV, CNRS, Université Paris-Saclay, IRD, Gif-sur-Yvette, France; ^2^Sorbonne Université, INRAE, CNRS, IRD, UPEC, Université Paris Cité, Institute of Ecology and Environmental Sciences of Paris (iEES-Paris), Paris, France

**Keywords:** calcium imaging, heterologous expression, odorant receptor, insect, *Drosophila melanogaster*, *Spodoptera littoralis*

## Abstract

The primary actors in the detection of olfactory information in insects are odorant receptors (ORs), transmembrane proteins expressed at the dendrites of olfactory sensory neurons (OSNs). In order to decode the insect olfactome, many studies focus on the deorphanization of ORs (i.e., identification of their ligand), using various approaches involving heterologous expression coupled to neurophysiological recordings. The “empty neuron system” of the fruit fly *Drosophila melanogaster* is an appreciable host for insect ORs, because it conserves the cellular environment of an OSN. Neural activity is usually recorded using labor-intensive electrophysiological approaches (single sensillum recordings, SSR). In this study, we establish a simple method for OR deorphanization using transcuticular calcium imaging (TCI) at the level of the fly antenna. As a proof of concept, we used two previously deorphanized ORs from the cotton leafworm *Spodoptera littoralis*, a specialist pheromone receptor and a generalist plant odor receptor. We demonstrate that by co-expressing the GCaMP6s/m calcium probes with the OR of interest, it is possible to measure robust odorant-induced responses under conventional microscopy conditions. The tuning breadth and sensitivity of ORs as revealed using TCI were similar to those measured using single sensillum recordings (SSR). We test and discuss the practical advantages of this method in terms of recording duration and the simultaneous testing of several insects.

## 1. Introduction

Olfaction is a predominant sense for most animals, which are exposed to a plethora of chemical cues in their environment, signaling food sources, conspecifics, mating partners or predators among others (Hansson, 1999; de Bruyne and Baker, 2008; Hansson and Stensmyr, 2011; Li and Liberles, 2015). While the visual system uses a few photoreceptors to decode the entire light spectrum, the olfactory system deploys a high diversity and plasticity in its structure in order to detect and discriminate thousands of discrete odorants differing in molecular structure and physicochemical properties (Keller and Vosshall, 2016). In insects, odorants are detected at the periphery by olfactory sensory neurons (OSNs) housed within cuticular hairs located mainly on the insect’s antennae or maxillary palps. OSNs detect odorants through chemoreceptors belonging to different families of transmembrane proteins, among which odorant receptors (ORs) play a major role (Joseph and Carlson, 2015; Robertson et al., 2019). ORs form odorant-gated ion channels (Sato et al., 2008; Wicher et al., 2008) in a heteromeric complex with a conserved co-receptor (ORco) (Larsson et al., 2004; Vosshall and Hansson, 2011). In addition to ORco, insects typically express a high number of OR genes, ranging from about 3 in some dragonflies to more than 500 in various ant species (Andersson et al., 2015; Yan et al., 2020). The generally accepted model obtained from work in *Drosophila melanogaster* is that only one OR gene is expressed together with ORco in a given OSN, and that all OSNs carrying a given OR project to the same glomerulus in the primary olfactory brain center, the antennal lobe (Couto et al., 2005; Pitts et al., 2016). Recently, some exceptions to this rule have been found, for instance in some drosophilid and mosquito species in which co-expression of different types of ORs, or of ORs together with IRs (ionotropic receptors) have been demonstrated (Ebrahim et al., 2015; Karner et al., 2015; Auer et al., 2022; Herre et al., 2022; Task et al., 2022). In addition, such a simple system does not seem to apply in locusts due to important differences in the number of ORs compared to the number of glomeruli (142 vs. 1,000) (Ignell et al., 2001; Chang et al., 2022).

Whatever the exact organization within a given insect species, its olfactory capacities generally depend on its OR gene repertoire and on the functional properties of the OR proteins, in particular their sensitivity and tuning breadth. Accordingly, the deorphanization (i.e., identification of the ligands) of ORs and the study of their functional properties have attracted strong interest in the last decades (Table 1). Interest for ORs and their ligands also stems from research aiming to develop new odorant biosensors that can be used as tools in commercial devices for the detection of volatile organic compounds (Kawano et al., 2011; Liu et al., 2013). Accordingly, purely artificial approaches have been developed, such as a synthetic OR is embedded in a bilayer lipid membrane on a chip, and electrical activity is recorded using a conventional patch-clamp system (Funakoshi et al., 2006; Osaki and Takeuchi, 2017; Misawa et al., 2019) or into systems that mimic the cell membrane as nanodiscs or nano-liposomes (Khadka et al., 2019; Murugathas et al., 2019). Such a system may be applied for insect OR deorphanization (Misawa et al., 2019). However, the most common approach used for deorphanization is heterologous expression of ORs within extant biological systems (Dobritsa et al., 2003; Kurtovic et al., 2007; Wanner et al., 2007; Montagné et al., 2012; Claudianos et al., 2014; de Fouchier et al., 2015, 2017). This involves the insertion of gDNA or cRNA encoding an OR of interest from one species into another species. Different types of *in vitro* and *in vivo* host systems have been used. As *in vitro* systems, HEK-293 cells (human embryonic kidney cells) are mostly used for the functional study of mammalian ORs (Krautwurst et al., 1998; Wetzel et al., 1999; Katada et al., 2003; Corcoran et al., 2014) but appear to be efficient for insect ORs also (Grosse-Wilde et al., 2006), while Sf9 cells (from a *Spodoptera frugiperda* ovary cell line) can be used to study insect ORs (Claudianos et al., 2014; Xu et al., 2015). OR activation by odorant molecules is then monitored by calcium imaging. Expression in *Xenopus* oocytes followed by electrophysiology recordings is also widely used, from mammals to insects (Wetzel et al., 1999; Wanner et al., 2007, 2010; Bavan et al., 2014; Peterlin et al., 2014; Wang et al., 2016; Hou et al., 2020). As an *in vivo* system, the “empty neuron system” of the fruit fly *Drosophila melanogaster* is an appreciable host for insect ORs, because it conserves the natural environment of an OSN, with the presence of odorant-binding proteins (OBPs) (Kruse et al., 2003; Xu et al., 2005), odorant-degrading enzymes (ODEs), accessory cell environment (trichogen, tormogen and thecogen cells) and membrane proteins such as Sensory Neuron Membrane Proteins (Thurm and Küppers, 1980; Steinbrecht, 1992; Cassau and Krieger, 2021; Prelic et al., 2022). The actual roles of each of these components are still debated but may be crucial for measuring relevant OR responses. Therefore, this system creates an optimal environment in which the probability of deorphanizing an OR is ultimately enhanced. One option involves replacing the endogenous odorant receptors OR22a and OR22b present within the ab3A neurons of large basiconic sensilla, with the OR of interest (Hallem and Carlson, 2006; de Fouchier et al., 2017; Chahda et al., 2019). Similarly, it is possible to replace the *Drosophila* OR67d receptor by an OR of interest within OSNs of the at1 trichoid sensilla (Kurtovic et al., 2007; Montagné et al., 2012; de Fouchier et al., 2015, 2017; Brand et al., 2020). Both systems have been used for the functional study of insect ORs, with the basiconic (ab3) system being preferentially applied for generalist receptors like OR22a (de Fouchier et al., 2017), and the trichoid system (at1) preferred for pheromone receptors like OR67d (Montagné et al., 2012). Beyond the expression of OR genes in *Drosophila* OSNs, OR deorphanization involves measuring the electric activity of the transformed neurons and testing a wide panel of odorants in order to find the correct ligands. This is usually performed using single-sensillum recordings (SSR) of ab3 or at1 sensilla (Dobritsa et al., 2003; Hallem et al., 2004; Kurtovic et al., 2007; Montagné et al., 2012; de Fouchier et al., 2017; Chahda et al., 2019). While well established and robust, the SSR approach has some limitations that may reduce its use for high throughput approaches needed for a large scale OR deorphanization. For instance, single sensillum electrical contact is relatively short lived, only one insect can be recorded at a time and recordings focus on one sensillum at a time. In this context, optical imaging techniques may offer interesting opportunities to obtain longer recording times and recordings from several insects simultaneously.

**Table 1:** Methods for ORs functional characterization by expression in heterologous system.

Functional characterization of ORs by expression in heterologous systems						
Expression system	System	Recording method	Odorant stimulation	Type of OR species	Screening capacity	References
*Xenopus* oocyte	*In vitro*	Patch-clamp	Liquid	Mammal/Insect/Human	Broad	1,2,3
Sf9	*In vitro*	cAMP response recordings	Liquid	Human	Broad	4
Sf9	*In vitro*	Calcium imaging	Liquid	Insect	Broad	5,6
Human embryonic kidney (HEK)-293T cells	*In vitro*	Patch-clamp	Liquid	Mammal/Insect	Broad	7,8
Human embryonic kidney (HEK)-293T cells	*In vitro*	Calcium imaging/Spectrofluorimetric calcium assay	Liquid	Mammal/Human	Broad	9,1
*Drosophila melanogaster* empty neuron systems	*In vivo*	Electrophysiology (SSR)	Volatile	Insect	Narrow	11,12,13,14,15,16
*Drosophila melanogaster* empty neuron systems	*In vivo*	Transcuticular calcium imaging	Volatile	Insect	Broad	This study
*E. coli* cell free	*In vitro*	Immunoaffinity chromatography	Liquid	Insect	Broad	17
Artificial membrane BLM	*In vitro*	Patch-clamp	Volatile/Liquid	Insect	Broad	18
Adenovirus recombined	*In vitro*	Calcium imaging	Liquid	Insect	Broad	19
HeLa/15	*In vitro*	Calcium imaging	Liquid	Mammal	Broad	20
HeLa/Olf	*In vitro*	Calcium imaging	Liquid	Mammal	Broad	20,21
*Drosophila* S2 cells	*In vitro*	Calcium imaging	Liquid	Insect	Broad	22
Bm5	*In vivo*	Behavioral assay	Liquid	Insect	Broad	23
High-five insect cells	*In vitro*	–	–	Insect	Broad	24
Wheat-germ cell-free expression system	*In vitro*	graphene field-effect transistor	Liquid	Mammal	Broad	25,26
Hana3A cells	*In vitro*	cAMP response recordings	Liquid	Mammal	Broad	27
*Cercopithecus aethiops* SV40 (COS)-7 cells	*In vitro*	Spectrofluorimetric calcium assay	Liquid	Mammal	Narrow	28
ODORA cells	*In vitro*	Spectrofluorimetric calcium assay	Liquid	Mammal	Narrow	29
Rabbit reticulocyte lysate	*In vitro*	–	–	Mammal	Broad	30

1, Wetzel et al. (1999); 2, Wanner et al. (2007); 3, Hou et al. (2020); 4, Parker et al. (1994); 5, Anderson et al. (2009); 6, Claudianos et al. (2014); 7, Grosse-Wilde et al. (2006); 8, Corcoran et al. (2014); 9, Hamana et al. (2010); 10, Gonzalez-Kristeller et al. (2015); 11, Dobritsa et al. (2003); 12, Hallem et al. (2004); 13, Kurtovic et al. (2007); 14, Montagné et al. (2012); 15, de Fouchier et al. (2017); 16, Chahda et al. (2019); 17, Tegler et al. (2015); 18, Misawa et al. (2019); 19, Zhao et al. (1998); 20, Shirokova et al. (2005); 21, Krautwurst (2008); 22, Lundin et al. (2007); 23, Sakurai et al. (2011); 24, Tsitoura et al. (2010); 25, Kaiser et al. (2008); 26, Yoshii et al. (2022); 27, Thach et al. (2017); 28, Levasseur et al. (2003); 29, Levasseur et al. (2004); 30, Ritz et al. (2013).

In the present study, we asked if optical imaging approaches can be used as an additional technique for OR deorphanization. Unlike most insects, the cuticle of the *Drosophila* antenna is translucent, so neurons can be imaged through the cuticle if a genetically encoded fluorescent protein sensor with a strong baseline fluorescence is used, an approach termed transcuticular calcium imaging (in this study, shorted as TCI) (Pelz et al., 2006; Kamikouchi et al., 2010; Strauch et al., 2014). To test this possibility, we used two previously characterized ORs from the cotton leafworm *Spodoptera littoralis* (Montagné et al., 2012; de Fouchier et al., 2015, 2017), a specific pheromone receptor (SlitOR6) expressed in at1 trichoid sensilla, and a generalist receptor (SlitOR29) expressed in ab3 basiconic sensilla. We showed that by co-expressing the strong baseline calcium sensors GCaMP6s/m with the OR of interest, it is possible to measure odor-evoked responses under conventional microscopy conditions. We then compared the responses evoked by these receptors using TCI with those previously measured using SSR (de Fouchier et al., 2017), focusing on their tuning breadth and sensitivity. We also tested the potential duration of TCI recordings and developed a 2-individual preparation for improved experimental throughput.

## 2. Materials and methods

### 2.1. *Drosophila* genetics

The generation of transgenic flies expressing SlitOR6 within at1 OSNs instead of the endogenous *Drosophila* receptor DmelOR67d (genotype *w; UAS-*Slit*OR6;* Dmel*OR67d^GAL4^*) was described previously (Montagné et al., 2012). To generate flies expressing SlitOR29 instead of DmelOR22a and OR22b in ab3A OSNs, the UAS-SlitOR29 line previously described (de Fouchier et al., 2017) was crossed to the mutant knock-in Dmel*OR22ab^GAL4^* line (kindly provided by Dr. John Carlson) (Chahda et al., 2019). Dmel*OR67d^GAL4^* and Dmel*OR22ab^GAL4^* lines were also crossed to lines expressing GCaMP6m (genotype *w; UAS-GCaMP6m;* Dmel*OR67d^GAL4^*) or GCaMP6s (w[1118]; PBac[y[+mDint2] w[+mC] = 20XUAS-IVS-GCaMP6s]VK00005, Janelia Research Campus, Ashburn, United-States), to generate flies expressing these calcium indicators in at1 or ab3A OSNs, respectively. Prior to calcium imaging experiments, flies expressing either SlitOR6 or SlitOR29 were crossed with flies carrying the GCaMP6m/s calcium indicator to generate heterozygous flies co-expressing both an OR and a calcium indicator (genotypes *w; UAS-*Slit*OR6/UAS-GCaMP6m;* Dmel*OR67d^GAL4^* and *w;* Dmel*OR22ab^GAL4^;* UAS-*Slit*OR29/*UAS-GCaMP6s*).

### 2.2. *Drosophila* preparation and calcium imaging recordings

**Figure 1:**
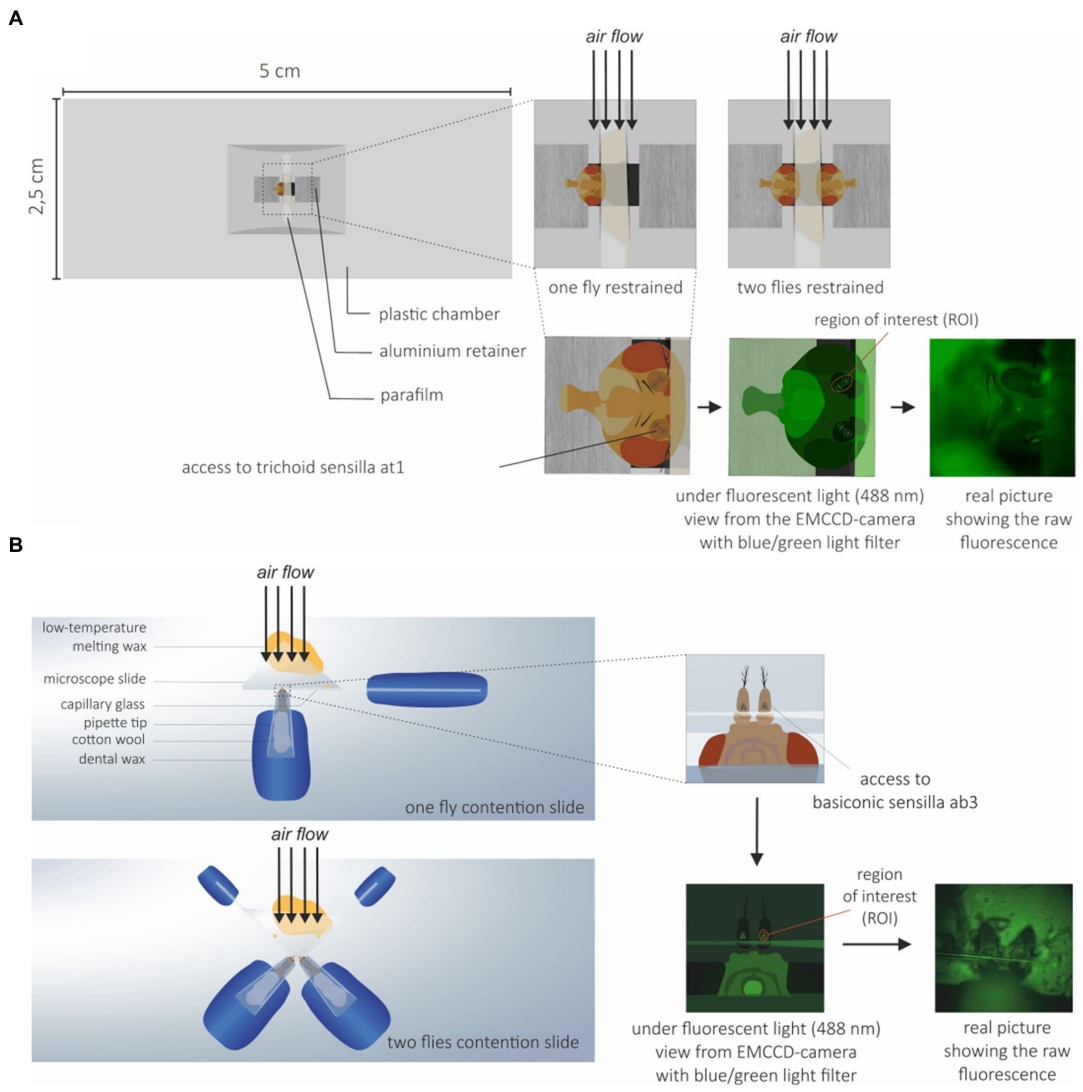
*Drosophila* preparation for calcium imaging. **(A)** Preparation for trichoid sensilla imaging: Flies were restrained to prevent movement during the experiments and to expose the trichoid at1 sensilla to the microscope objective and the EMCCD-camera. To increase the throughput of each experiment, a system with two flies on the same holder was also used. **(B)** Preparation for basiconic sensilla imaging: Flies were restrained to expose the basiconic sensilla to the microscope objective and EMCCD-camera. A holder with two flies was also used.

The flies used in calcium imaging experiments were 3–7 days old. Since different sets of OSNs were imaged on the fly antenna for the SlitOR6 and SlitOR29 experiments, two different preparations were used. For the SlitOR6 experiment, flies were immobilized in an ABS (Acrylonitrile-butadiene-styrene) plastic chamber with only the head protruding. Wings were attached using myristic acid to avoid fly body movement. The fly antennae were constrained with a thin piece of Parafilm^®^ (Bemis Company, Inc., Zurich) in order to expose the region of the antennae where at1 sensilla are located (Figure 1A; Pelz et al., 2006; Lin and Potter, 2015). For the SlitOR29 experiment, flies were restrained in a 200 μL plastic pipette tip, so that the antennae and half of the head protruded. The tip was attached on a microscope glass slide using dental wax, with the ventral side of the *Drosophila* facing upward. The antennae were then placed on a piece of microscope slide, and restrained by pressing a glass capillary between the second (pedicel) and the third segment (funiculus) of the antennae. The glass capillary was attached to the slide using low temperature melting wax to ensure that the antennae did not move during the experiment (Figure 1B). In order to increase the throughput of each experiment, two-fly holders were also made for both systems (Figures 1A,B).

Calcium imaging recordings were performed with a 10x water-immersion objective (Olympus UMPlanFI 10x/0.30 W) on an epifluorescence microscope (Olympus BX-51WI) coupled to an EMCCD-camera (Evolve™ 512, Photometrics). Recordings were performed using dedicated routines under the Visiview 3.3.0.0 software. Excitation light at 488 nm was produced with a monochromator (Polychrome 5000). Each recording consisted in 100 frames sampled at a frequency of 5 Hz (20 s recording).

### 2.3. Olfactory stimulations

A constant airstream (3.5 L/min) was directed at a distance of 1 cm toward the fly’s antennae. It was composed of a main air flow of 3 L/min and of a secondary air flow of 500 mL/min. The secondary air flow could be directed to one of two sub-circuits (one containing an odorant source and another without any odorant) before being reinjected into the main airflow. Most of the time, air flowed through the odorless sub-circuit. Olfactory stimulation was triggered by the imaging computer, redirecting the secondary flow to the odorant sub-circuit. The olfactory stimulation lasted for 1 s, starting at the 15th frame (i.e., after 3 s) and lasting until the 20th frame.

The odorant sub-circuit used interchangeable stimulation cartridges containing the odor sources (see below). The other sub-circuit included an identical cartridge without odorant. An air extractor, placed behind the bee prevented odorant accumulation.

The stimulation cartridges consisted of Pasteur pipettes each containing a 1 cm^2^ piece of filter paper loaded with 5 μL of the odorant solution, and a 1 mL plastic pipette tip at the back to close the cartridge. New stimulation cartridges were prepared every day. A pipette containing a piece of filter paper soaked with solvent alone was used as control stimulus.

Prior to each experiment, we tested the absence of response to the ligands of the endogenous DmelOR as a negative control. Potential flies that potentially respond to the negative control were evicted from the experiments. We used cis-vaccenyl acetate (cVA) as the ligand for DmelOR67d, and ethyl hexanoate as the ligand for DmelOR22a.

We tested 4 different odorants on SlitOR6, chosen from de Fouchier et al. (2017): 1-hexanol, benzaldehyde, linalool and the *S. littoralis* pheromone component (*Z,E*)-9,12-tetradecadienyl acetate (further referred to as (*Z,E*)-9,12-14:OAc). For SlitOR29, 13 odorants were used based on their various response levels in de Fouchier et al. (2017): 1-heptanol, 1-hexanol, 1-indanone, (E)-2-hexenol, acetophenone, benzaldehyde, geraniol, (E)-2-hexenal, (Z)-3-hexenyl acetate, β-myrcene, (E)-ocimene, sulcatone and (*Z,E*)-9,12-14:OAc. The moth pheromone (*Z,E*)-9,12-14:OAc was diluted in hexane at 1 μg.μL^−1^ (10 μg on the filter paper). The other odorants were diluted at 10 μg.μL^−1^ (100 μg on the filter paper) in mineral oil, except for 1-indanone which was diluted in ethanol. The respective solvents were used as controls. Odorants were presented in a random order except in the case of dose–response experiments in which odorants were tested in increasing doses. The pheromone was tested at doses ranging from 100 pg. to 10 μg, (E)-ocimene was tested from 100 pg. to 100 μg, and (Z)-3-hexenyl acetate from 100 ng to 100 μg.

For the two-fly experiment and the repeated stimulation experiment, we used the most potent ligand of SlitOR29, (E)-ocimene at 100 μg on the filter paper. Stimulations were applied every 2 min for the repeated stimulation experiment, until responses stopped. The maximum number of stimulations applied was 255. Because the experiment could not be carried out for such a long time for all individuals, the average curve presented in the results is based on the first 180 stimulations of each fly.

In addition to recordings with odorants and their solvents, we also performed a recording without any stimulus, in order to measure the decay of GCamp6 fluorescence during the 100 frames of a recording.

### 2.4. Data analysis and statistics

The calcium imaging data were extracted using VisiView 3.3.0.0 software. Data processing was performed using custom scripts in R software (v3.4.3) and Microsoft Excel 2013. Regions of interest (ROI) were drawn around each antenna. The average fluorescence level observed within each ROI at each frame was exported (100 frames per stimulation). To obtain fluorescence changes over time, we calculated ΔF/F_0_ = (F-F_0_)/F_0,_ where F_0_ is the mean fluorescence value over 5 frames before the stimulation (which started 3 s after the beginning of the recording, between the frame 16–20) and F the fluorescence at frame n. To correct for photobleaching, the curve measuring GCaMP6 fluorescence decay over time was subtracted from all other curves.

As calcium signals are slow signals, response amplitude was generally calculated as the average of 15 frames (3 s) after the start of the stimulation (frames 17–31). In the case of SlitOR29, both SSR and TCI provided a range of strong and weaker ligands, offering us the opportunity to perform a temporal analysis asking when the calcium signal was the best fit for the SSR data (measured very shortly after the stimulus—see results). To do that, we performed a cross-correlation using the calcium signal for each odorant at each time frame.

Statistical analysis was performed with GraphPad Prism 7 (GraphPad Software Inc.). The normal distribution of all data was tested using the Shapiro–Wilk normality test. Response amplitudes to the different stimuli were compared using the Friedman test, as the data did not consistently follow a normal distribution. When significant, the response to each odorant was compared to its solvent control using Dunn’s multiple comparison test. Pearson’s correlations were applied for correlation analyses between SSR and TCI data (raw SSR data were retrieved from De Fouchier et al., 2017).

The sparseness of OR response spectra was calculated using the formula from Rolls and Tovee (1995):


$$S=\left(\frac{1}{1-\frac{1}{n}}\right)\times \left(1-\frac{\left(\sum_{i=1,n}r_{i}/n\right)2}{\sum_{i=1,n}\left(r_{i}^{2}/n\right)}\right)$$

With *r_i_* being the amplitude of the response to the stimulus *i* in the set of *n* stimuli. As this formula does not allow the calculation of negative responses, these were set to 0.

For the dose–response analysis, we compared response amplitudes obtained for the different doses using ANOVA for repeated measurements with the Greenhouse–Geisser correction, after verifying the normal distribution of the data using the Shapiro–Wilk test. Pairwise comparisons between doses and their corresponding negative control were performed using the Dunnett’s multiple comparison test.

We also compared the normalized dose–response curves between SSR and TCI experiments. Normalized curves were established using the highest response as 100% and the lowest response (to solvent) as 0%. Comparisons of EC_50_ and Hill slope coefficients were performed using the Mann–Whitney U test.

For comparisons of response amplitudes between one-individual and two-individual restraining experiments, an unpaired t test was used. Lastly, for the repeated stimulation experiment, a Friedman test was used to compare responses amplitude in the course of experiment. Multiple comparisons of response amplitudes between the control and any particular stimulation were performed using Dunn’s multiple comparison test.

## 3. Results

In this study, we investigated whether TCI can be an interesting alternative to SSR for the functional study of odorant receptors (ORs) expressed in a heterologous system, the olfactory system of *Drosophila melanogaster*. We chose to test two different ORs of *Spodoptera littoralis* (Montagné et al., 2012; de Fouchier et al., 2015, 2017) expressed in the two types of neurons largely used for OR studies: a narrowly tuned pheromone receptor (SlitOR6) expressed in the at1 neurons of trichoid sensilla, and a broadly tuned plant volatile receptor (SlitOR29) expressed in the ab3A neurons of basiconic sensilla.

### 3.1. Imaging the activity of a pheromone receptor in at1 sensilla

**Figure 2:**
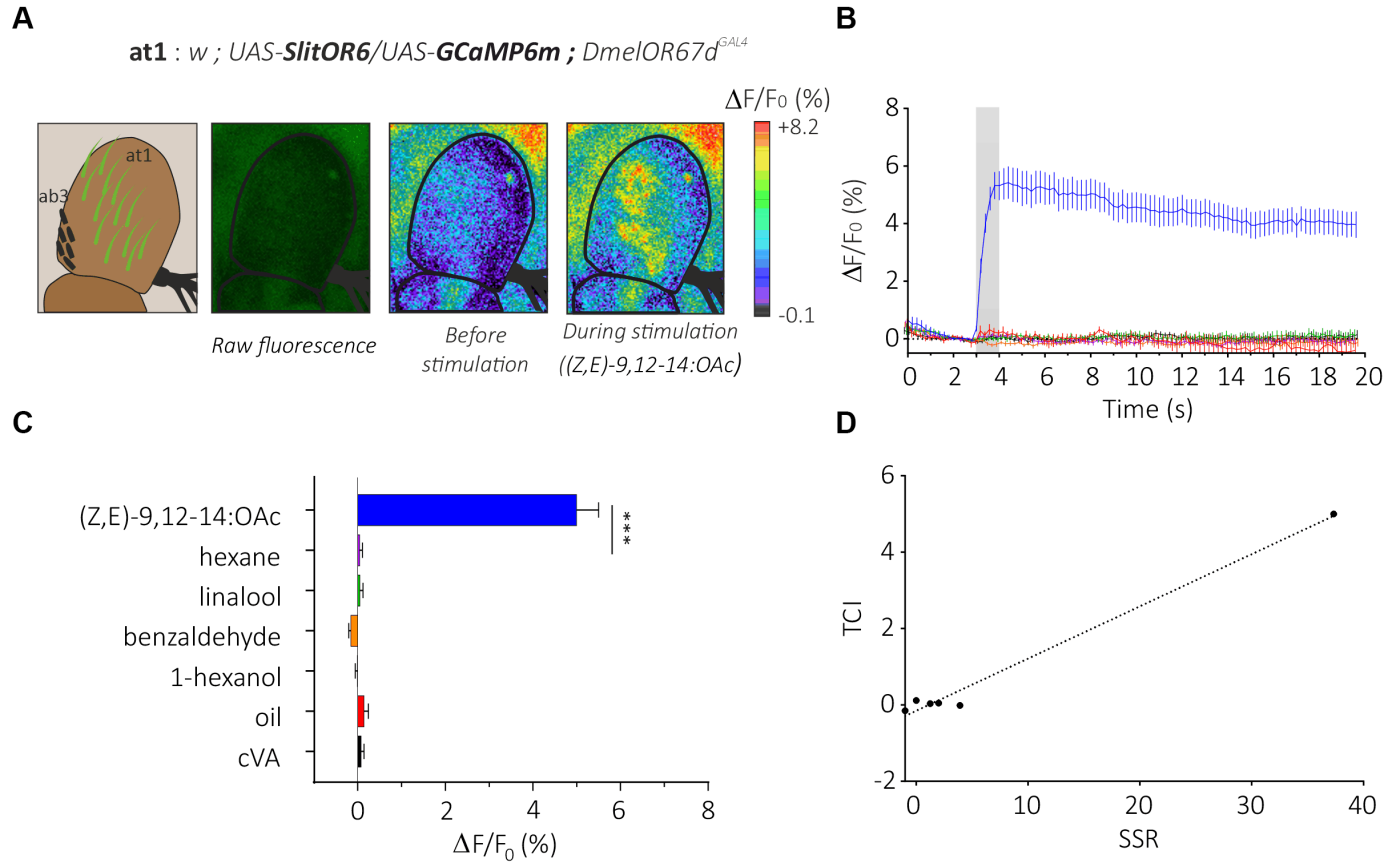
Analysis of the moth pheromone receptor SlitOR29 by transcuticular calcium imaging from at1 sensilla. **(A)** From left to right, representation of at1 trichoid and ab3 basiconic sensilla on the third segment of the *Drosophila* antenna; raw fluorescence image and calcium activity maps before and during stimulation with the moth pheromone (*Z,E*)-9,12-14:OAc. **(B)** Time course of calcium signals for the different stimuli (mean ± SEM, *n* = 16). Odorant stimulation is represented by the gray bar. **(C)** Amplitude of SlitOR6 calcium responses from at1 OSNs (mean ± SEM). Responses to the odorants were compared to their respective controls (hexane for the moth pheromone, and mineral oil for other odorants) (*n* = 16, ***p < 0.001). **(D)** Representation of TCI data as a function of SSR data (retrieved from De Fouchier et al., 2017) for the SlitOR6 experiment.

We first generated flies simultaneously expressing SlitOR6 and the calcium probe GCaMP6m in neurons of the at1 sensilla (*w*; *UAS-SlitOR6/UAS-GCaMP6m; DmelOR67d^GAL4^*, Figure 2A). In these flies, we observed a clear pattern of calcium responses to the different stimuli we presented (*n* = 16, Friedman’s test: *Q* = 8.25, *p* < 0.001). As expected, none of these flies responded to cVA, the ligand of the endogenous receptor (negative control). The SlitOR6 ligand (*Z,E*)-9,12-14:OAc induced strong calcium signals (4.99 ± 0.50%, Dunn’s test, *p* < 0.0001) in the region of the antenna where the at1 sensilla are located (Figures 2A–C). Likewise, as this receptor is very specific, we observed no significant response to the three other odorants and to the solvent controls (mineral oil for the three plant volatiles and hexane for the pheromone). As an additional control, we tested the same odorant panel on a control genetic line which expresses the calcium probe but not the moth receptor (*w*; *UAS-GCaMP6m; DmelOR67d^GAL4^*) and observed no significant responses (*n* = 16, Friedman’s test, *Q* = 2.14, *p* = 0.82; Supplementary Figure S1A).

This response pattern is perfectly in line with that obtained by de Fouchier et al. (2017) using SSR on SlitOR6-expressing at1 sensilla. Consequently, our antenna imaging data and the SSR data from de Fouchier et al. (2017) are strongly similar, although we did not perform any correlation analysis due to the low number of correlation point (Figure 2D). Calculation of the sparseness of this receptor based on the distribution of response amplitudes observed in TCI yielded a very high value (*S* = 0.98) identical to that obtained with SSR (de Fouchier et al., 2017). Thus, TCI and SSR provided a similar response pattern from SlitOR6-expressing at1 neurons.

**Figure 3:**
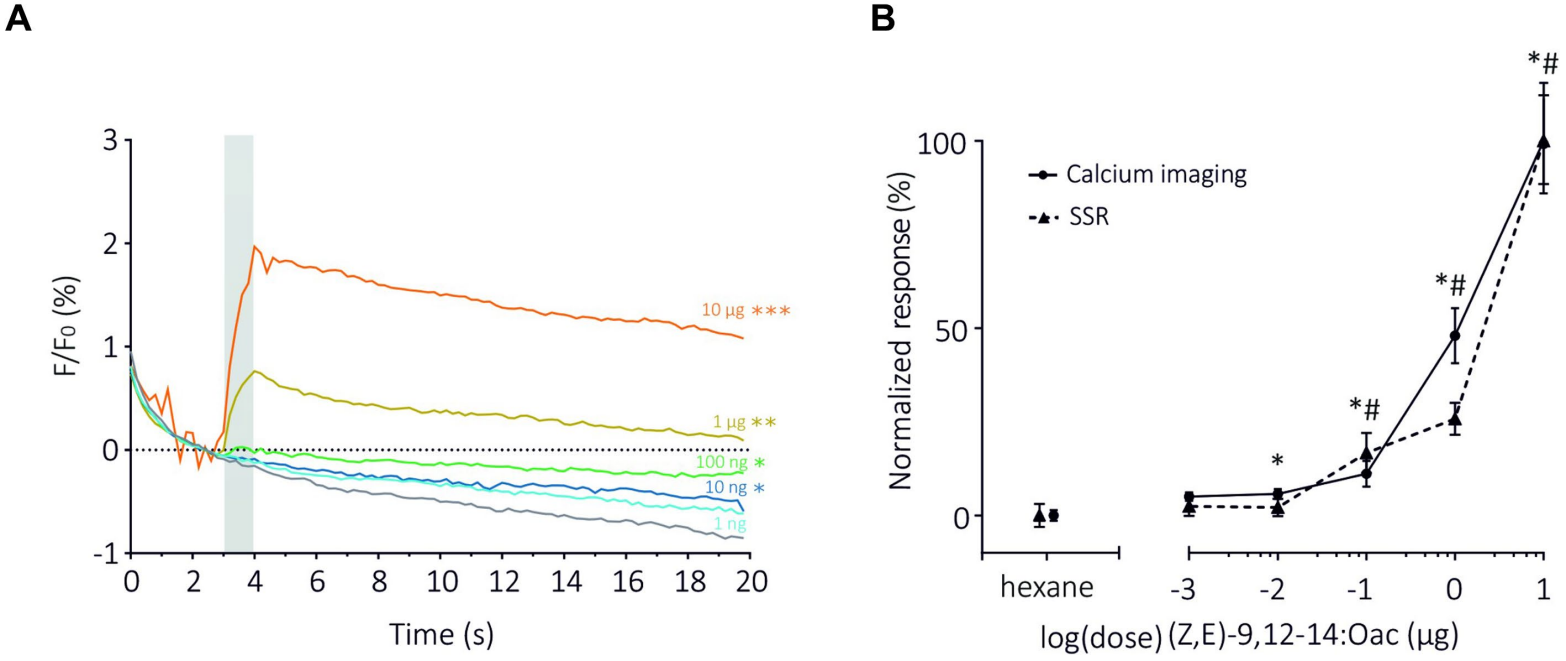
Dose–response analysis of SlitOR6 between TCI and SSR. **(A)** Time course of dose–response analysis of SlitOR6 in calcium imaging with increasing doses of the moth pheromone (*Z,E*)-9,12-14:OAc (*n* = 16, *p < 0.01, **p < 0.001, ***p < 0.0001). Odorant stimulation is represented by the gray bar, doses are represented by colors from light blue to orange (light blue: 1 ng; dark blue: 10 ng; green: 100 ng; yellow: 1 μg; orange: 10 μg; gray: solvent control). **(B)** Comparison of normalized responses (%) to increasing doses of (*Z,E*)-9,12-14:OAc between TCI (plain line, *n* = 11, *p < 0.05) and SSR (dashed line, *n* = 5, #*p* < 0.05) (data from de Fouchier et al., 2017) (Rm-ANOVA and *post-hoc* Dunnett’s test).

Next, we compared the two methods in terms of sensitivity by focusing on the dose–response analysis of SlitOR6 to the pheromone (*Z,E*)-9,12-14:OAc (de Fouchier et al., 2017; Figure 3). As expected, with both methods, the amplitude of the response increased significantly with the quantity of odorant. Using TCI, we observed that the response started to be significant at a pheromone dose of 10 ng (*n* = 15, Repeated measure ANOVA, *F*_1.378_ = 37.36, Greenhouse–Geisser’s *ε* = 0.23, *p* < 0.001, *post hoc* Dunnett’s multiple comparison test *p* = 0.042, Figure 3A). Under similar conditions, this threshold reached 100 ng in the SSR experiments (de Fouchier et al., 2017). Despite this difference, the dose–response curves obtained with the two methods appeared very similar. To be able to compare the shapes and EC_50_ of the curves, they were normalized (Figure 3B). We found comparable EC_50_ in both systems, 0.96 ± 0.14 μg for TCI and 0.10 ± 0.02 μg for SSR (Mann–Whitney U Test, *U* = 11, *p* = 0.07). The steepness of each curve, characterized by the Hill Slope, was similar between the methods (1.82 ± 0.45 for TCI and 1.77 ± 0.98 for SSR, Mann–Whitney U test; *U* = 34, *p* = 0.80).

### 3.2. Imaging the activity of a generalist receptor in ab3 sensilla

**Figure 4:**
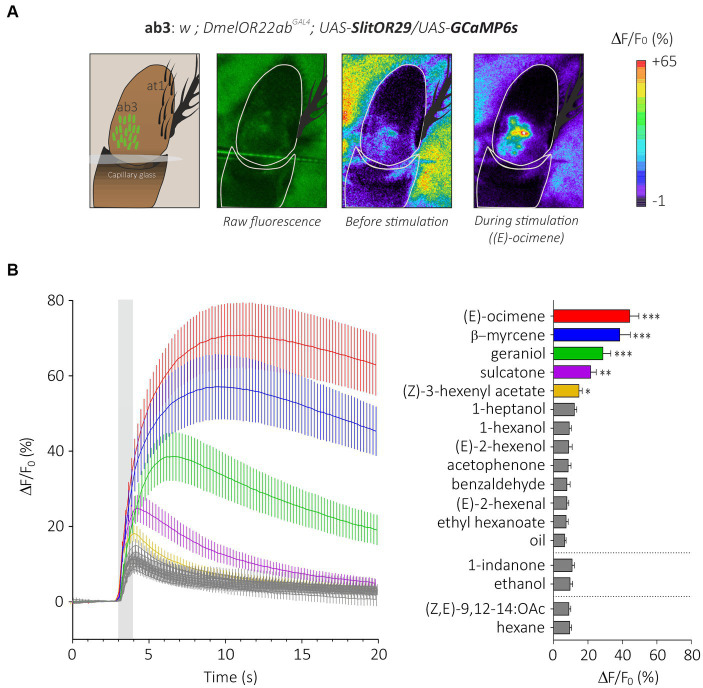
Analysis of the generalist moth receptor SlitOR29 by transcuticular calcium imaging from ab3 sensilla. **(A)** From left to right, a representation of the at1 trichoid and the basiconic ab3 sensilla expressing the calcium probe GCaMP6s on the third segment of the *Drosophila* antenna; raw fluorescence image and calcium activity maps before and during stimulation with (E)-ocimene. **(B)** Left: time course of calcium signals for the different stimuli (mean ± SEM, *n* = 16). Odorant stimulation is represented by the gray bar. Right: Amplitude calcium responses of SlitOR29 expressed in the fly ab3 sensilla (mean ± SEM). Responses were compared to control (hexane for the moth pheromone, ethanol for 1-indanone and mineral oil for the other odorants) (*n* = 16, ***p < 0.0001, **p < 0.001, *p < 0.01).

We expressed the generalist odorant receptor SlitOR29 in ab3A neurons of *Drosophila melanogaster* simultaneously with the calcium indicator GCaMP6s. We thus generated transgenic flies with the genotype *w;DmelOR22ab^GAL4^;UAS-SlitOR29/UAS-GCaMP6s*. In these flies, we observed a clear pattern of calcium responses to the different stimuli we presented (Figures 4A,B, *N* = 16, Friedman test, *Q* = 118.8, *p* < 0.0001). As expected, none of the flies responded to the ligand of DmelOR22a, ethyl hexanoate (*post hoc* Dunn’s multiple comparison test, *p* > 0.99). Based on the SSR results from de Fouchier et al. (2017), we tested some of the most potent ligands of SlitOR29: (E)-ocimene, β-myrcene, geraniol, sulcatone (terpenes) and (Z)-3-hexenyl acetate (aliphatic ester), but also other odorants that did not trigger responses from SlitOR29 previously: (E)-2-hexenal, (E)-2-hexenol, 1-hexanol, 1-heptanol, (Z,E)-9,12-14:OAc (aliphatics), benzaldehyde, acetophenone, 1-indanone (aromatics) and linalool (terpenes). As observed in the previous SSR experiments, (E)-ocimene and β-myrcene induced the highest activity in ab3A neurons expressing SlitOR29, with amplitudes of 44.23 ± 5.31% and 38.46 ± 6.16%, respectively (*post hoc* Dunn’s multiple comparison test, *p* < 0.0001). Geraniol (28.70 ± 4.55%), sulcatone (21.55 ± 3.28%) and Z-(3)-hexenyl acetate (14.78 ± 1.91%) also elicited significant responses (Dunn’s test, *p* < 0.01). The other stimuli did not elicit any significant response (between 6.49 ± 0.84% and 12.1 ± 1.40%, *post hoc* Dunn’s multiple comparison test, *p* > 0.0081).

In contrast to flies carrying SlitOR29, small responses were observed in genetic control flies *w*;*DmelOR22ab^GAL4^;UAS-GCaMP6s* (Friedman’s test, *Q* = 118.2, *p* < 0.0001). Responses were significant for 1-hexanol (*p* = 0.0001), sulcatone (*p* = 0.005), (E)-2-hexenol (*p* = 0.007) and 1-heptanol (*p* = 0.0002) (Supplementary Figure S1B). Such small responses were previously observed from the same genetic control lines using SSR (Chahda et al., 2019). Note that these responses (<10%) were much lower than those observed with the ligands of SlitOR29 (>40%). In addition, the pattern of responses observed with the genetic control did not correlate with SSR responses of the SlitOR29 expressing line (Supplementary Figures S1C,D).

**Figure 5:**
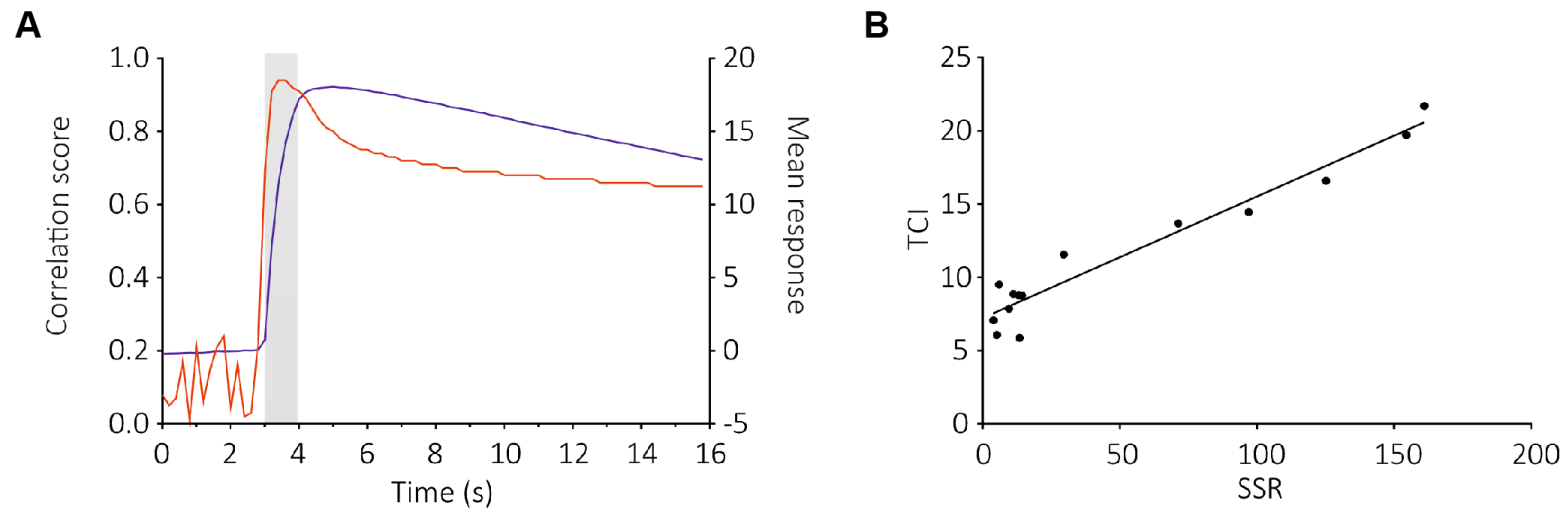
Cross-correlation analysis between TCI (measured at each time frame) and SSR data for the SlitOR29 experiment. **(A)** Pearson correlation between the amplitudes of calcium responses to the 14 ligands in **(B)** at each time frame throughout a recording and the amplitudes of the SSR response (red curve, left Y axis); On the same graph, the average response measured to the different ligands of SlitOR29 is given (blue curve, right Y axis). The correlation between SSR and TCI data is very high very early upon odor delivery, at a time when the calcium signal is still low. **(B)** Correlation between TCI data (measured 600 ms after odor onset—frame 18) and SSR data (Pearson correlation: *r*^2^ = 0.94, 12 df, *p* < 0.0001).

To compare these results with SSR data (De Fouchier et al., 2017), we used a correlation analysis between the two datasets. We observed a strong correlation between SlitOR29 responses to odorants using the TCI and SSR methods (Pearson correlation, *r*^2^ = 0.84, *p* < 0.0001, Figures 5A,B). This analysis used a measure of calcium signal amplitude that extended for 3 s, starting 400 ms after odor onset. In contrast, the SSR data were measured for only 500 ms, starting 100 ms after odor onset (De Fouchier et al., 2017). Since SlitOR29 offered a graded response from different ligands, a more detailed analysis of the correlation between SSR and TCI data was possible. We thus asked at which point in time the correlation between SSR and TCI data was the highest, by performing a cross-correlation of SSR data with the calcium data measured at each time frame. Figure 5 shows that, as expected, the correlation was very low when using calcium data measured before the stimulus, but it increased very quickly upon odor delivery, reaching a value of *r*^2^ = 0.91 already 400 ms after odor onset (frame 17), i.e., at a time when the calcium signal was only about 40% of its maximum. It reached *r*^2^ = 0.94 between 600 and 800 ms after odor onset. After odor delivery, the correlation tended to decrease but remained at a high level, around *r*^2^ = 0.80. Thus, TCI provides a faithful description of the responses recorded using SSR, and of the ligands of SlitOR29.

Lastly, to evaluate the tuning breadth of the receptor as measured using TCI, we computed its sparseness value. We obtained a low sparseness (*S* = 0.38), which is typical for generalist odorant receptors (Rolls and Tovee, 1995). The odorant panel tested here was slightly different from the panel tested in de Fouchier et al. (2017), so the direct statistical comparison of sparseness values is not applicable, but values correspond to a broad spectrum in both cases (*S* = 0.65 in SSR).

**Figure 6:**
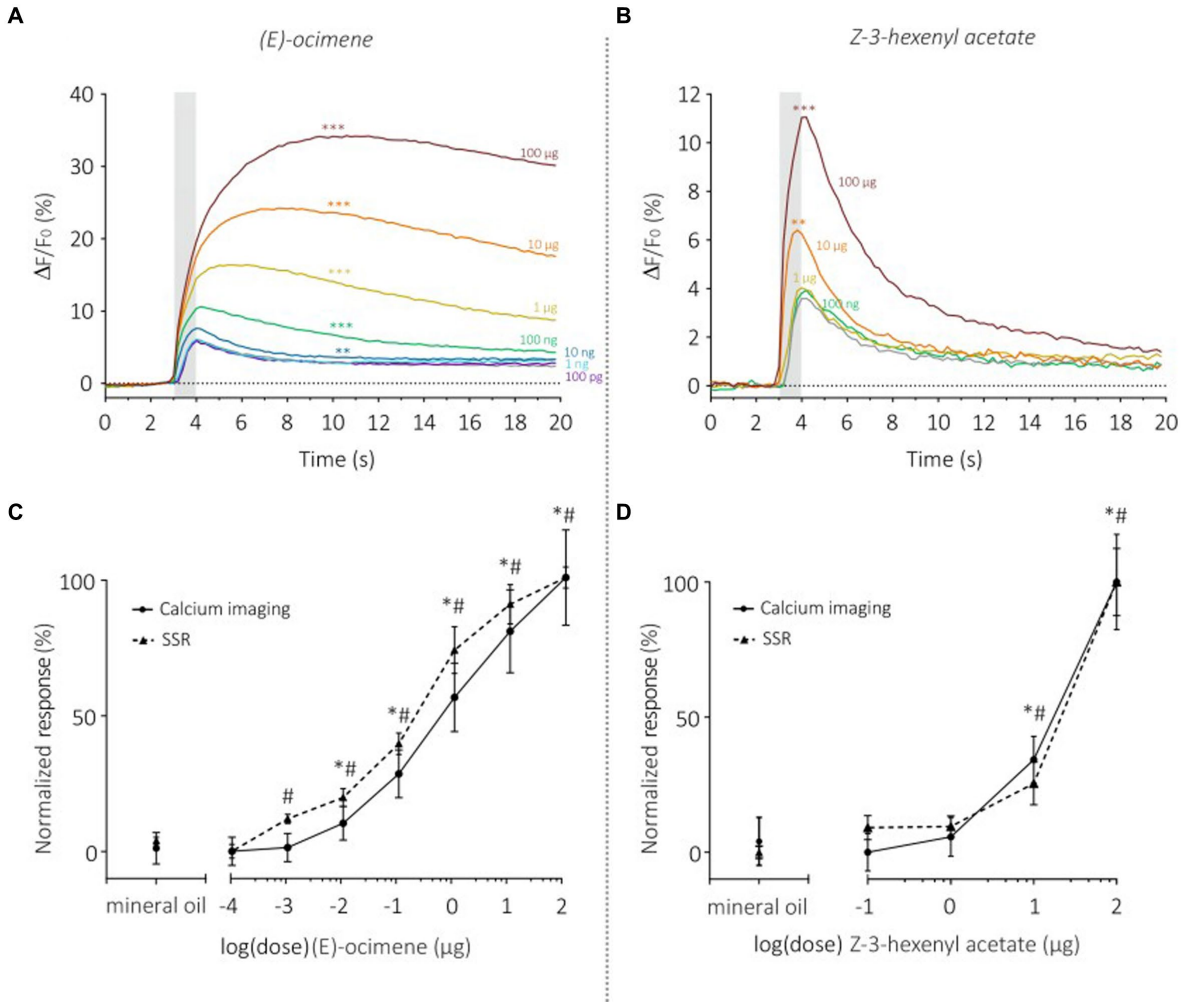
Dose–response analysis of SlitOR29 between TCI and SSR. Time course of dose–response analysis for (E)-ocimene in calcium imaging (*n* = 11, **p < 0.001, ***p < 0.0001) **(A)** and for Z-3-hexenyl acetate in calcium imaging (*n* = 11, **p < 0.001, ***p < 0.0001) **(B)**. Odorant stimulations are represented by colors from purple to dark red (purple: 100 pg.; light blue: 1 ng; dark blue: 10 ng; green: 100 ng; yellow: 1 μg; orange: 10 μg; dark red: 100 μg; gray: solvent control). **(C)** Comparison of normalized (E)-ocimene dose–response curves between TCI (plain line, *n* = 11, *p < 0.05) and SSR (dashed line, *n* = 5, #*p* < 0.05) (data from de Fouchier et al., 2017) (Rm-ANOVA and *post-hoc* Dunnett’s test). **(D)** Comparison of normalized responses to increasing doses of Z-3-hexenyl acetate between TCI (plain line, *n* = 11, *p < 0.05) and SSR (dashed line, *n* = 5, #*p* < 0.05) (data from de Fouchier et al., 2017) (Rm-ANOVA and *post-hoc* Dunnett’s test).

We then monitored the sensitivity of SlitOR29 using a dose–response analysis for two odorants, (E)-ocimene, the best ligand (Figure 6A), and (Z)-3-hexenyl acetate, the ligand that triggered the lowest significant response (Figure 6B). Dose–response analysis of (E)-ocimene showed an increase in response amplitude with increasing doses (Figure 6A). In TCI, the response amplitude became significant compared to the control at 10 ng (Repeated measure ANOVA, *F*_1.117_ = 58.37, *p* < 0.0001, *post hoc* Dunnett’s multiple comparison test, 10 ng vs. mineral oil, *p* < 0.001) whereas this occurred at 1 ng for SSR. Despite this slight difference in sensitivity threshold, the dose–response curves had similar shapes and slopes (Figure 6C). Indeed, we found no significant difference between the EC_50_ (TCI: 1.27 ± 0.37 μg; SSR: 0.59 ± 0.25 μg, Mann–Whitney U Test, *U* = 19 *p* = 0.08) or the Hill slope coefficient (TCI: 0.49 ± 0.04; SSR: 0.51 ± 0.07, Mann–Whitney U Test, *U* = 19 *p* = 0.08) measured on the standardized curves obtained for the two data sets. Similar results were observed for the dose–response analysis of (Z)-3-hexenyl acetate (Figure 6D) (Repeated measure ANOVA, *F*_1.317_ = 51.7, Greenhouse–Geisser’s *ε* = 0.32, *p* < 0.0001). A significant response was observed starting from a dose of 10 μg (Dunnett’s multiple comparison test, *p* = 0.001), the same dose as observed in SSR (De Fouchier et al., 2017). In order to compare TCI and SSR data, we analyzed the normalized curves using the EC_50_ and Hill slope coefficient (Figure 6D). We found similar EC_50_ for both curves (TCI: 11.52 ± 0.70 μg; SSR: 15.26 ± 2.65 μg, Mann–Whitney U test, *U* = 22, *p* = 0.07). The slope of the sigmoid curves, estimated using the Hill coefficient, was also similar with the two techniques (TCI: 4.14 ± 1.19; SSR: 4.41 ± 1.56, Mann–Whitney U Test, U = 39 *p* = 0.71). As above, very high correlations between TCI and SSR data were obtained for both (E)-ocimene (*r*^2^ = 0.98), and (Z)-3-hexenyl acetate dose–response curves (*r*^2^ = 0.97).

### 3.3. Maximizing experimental throughput

**Figure 7:**
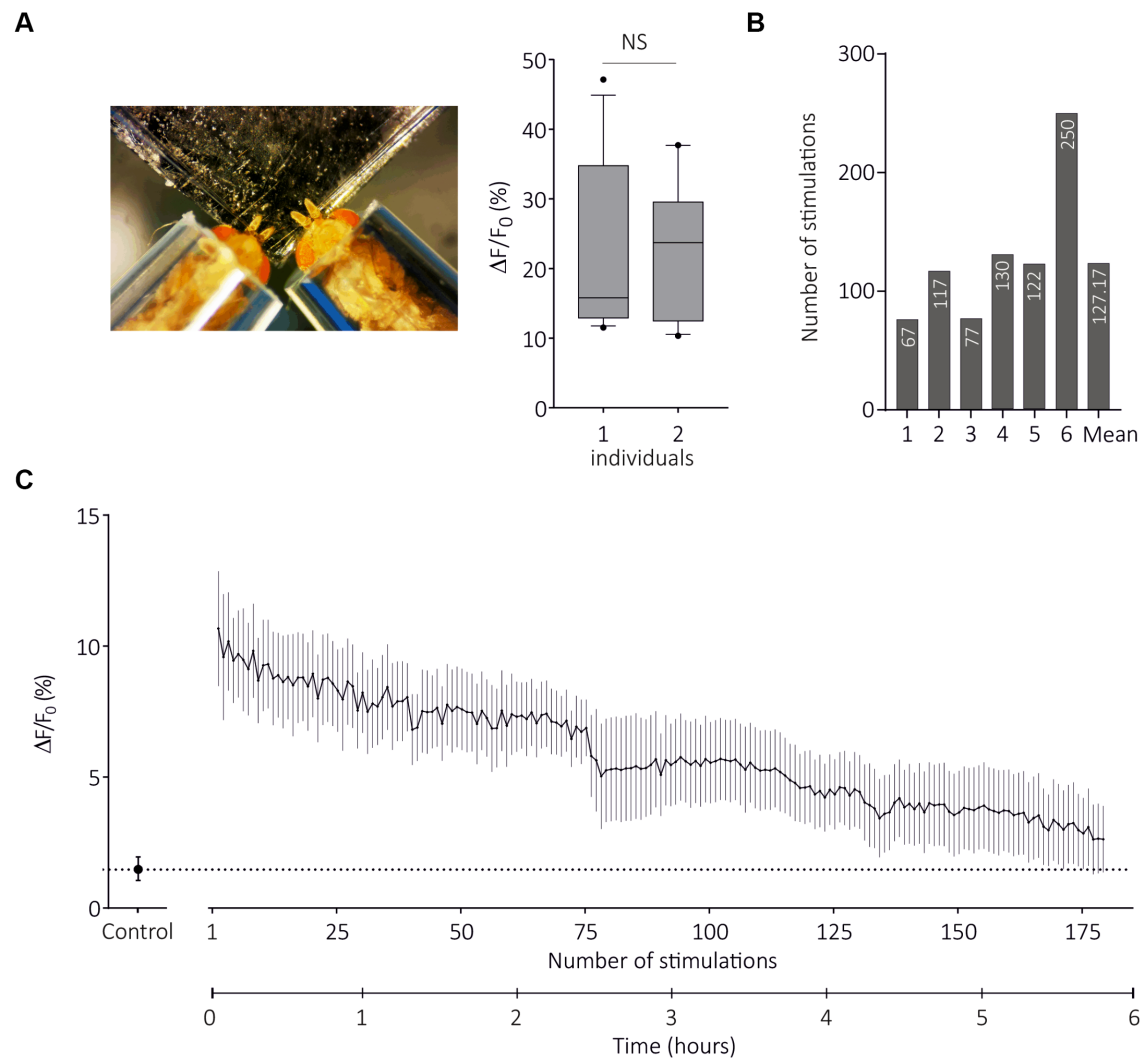
Transcuticular calcium imaging on multiple flies and for long durations. **(A)** From left to right, photograph of restraint with two flies expressing SlitOR29 in OSNs of ab3 sensilla; Boxplot (whiskers are 10–90 percentiles) showing amplitudes of responses to E-ocimene when flies are restrained alone (one individual, *n* = 7) or in pairs (two individuals, *n* = 10) (unpaired t test, *NS* = non-significant). **(B)** Number of stimulations for each fly until response amplitude decreased to 50% of the maximum response (*n* = 6). The mean value for the six individuals is shown at the end. **(C)** Amplitudes of calcium responses (mean ± SEM) to (E)-ocimene, every 2 min until responses stopped (*n* = 6). The control is the solvent (mineral oil).

As the TCI method has been validated by the previous experiments, we questioned its possible advantages over SSR. As optical imaging does not require the use of electrodes, it is in principle possible to image several individuals at the same time. We therefore developed a contention system that allows two flies to be imaged at the same time, taking care that both flies receive the same airflow (Figures 1A,B). For this experiment, we used SlitOR29 expressed in basiconic sensilla, although both basiconic and trichoid sensillum types and their respective two-fly contention systems can be used. We found that response amplitude to (E)-ocimene, the main ligand of SlitOR29, was identical in flies tested in the two-individual system as in flies tested alone (Figure 7A, one individual: *n* = 7, two individuals: *n* = 10, Unpaired t test, *t*_18_ = 0.1853, *p* = 0.85). Thus, this technique can be used to double the experimental throughput.

A possible limitation of SSR approaches may be the number of stimulations performed per sensillum and/or per individual. We next used the two-fly contention system and SlitOR29 expressed in basiconic sensilla to evaluate the maximal recording duration in TCI. We presented first the solvent and then (E)-ocimene every 2 min, until the responses stopped, with a maximum of 255 stimulations for one individual (*n* = 6, Figure 7B). Figure 7C presents the evolution of response amplitude (mean ± SEM) during 180 stimulations, which was the minimum number applied to all individuals. On average, the number of stimulations for which we still observed 50% of maximum response was 127 (Figure 7B). We observed a significant response after 5 h and 45 min of repeated stimulations on average (with a maximum of 8 h and 30 min). Note that the same odorant stimulation was repeated here, so olfactory adaptation may have occurred. The actual longevity of the responses might thus be even longer. We conclude from these experiments that TCI can be performed with several individuals at the same time, and allows for long recording times.

## 4. Discussion

The faster we deorphanize and characterize the odorant receptors of a given species, the more we will be able to understand which odorants are involved in the ecology of that insect, how it detects and processes them and thus, fundamentally, how it interacts with its olfactory environment. Today, the most common way to isolate ORs and to deorphanize them is via heterologous expression and electrophysiological recordings like SSR, which require an extreme immobility of the sample to measure neuronal activity and may suffer from short preparation duration (de Bruyne et al., 2001; Benton and Dahanukar, 2011; Lin and Potter, 2015). Here, we present a complementary approach for deorphanizing ORs. TCI may represent an efficient solution for screening large panels of odorants, before subsequently refining the study of a receptor’s ligands with techniques like SSR (Table 1).

We tested this technique in two different olfactory environments depending on the type of OR considered: the specific pheromone receptor SlitOR6 was expressed in at1 neurons instead of DmelOR67d while the receptor SlitOR29, which responds to a range of volatile plant compounds, was expressed in ab3A neurons instead of DmelOR22ab (Kurtovic et al., 2007; de Fouchier et al., 2017; Chahda et al., 2019). First, the response profiles recorded using TCI were very similar to those previously described using SSR. SlitOR6 was highly specific and responded strongly to its expected ligand, (*Z,E*)-9,12-14:OAc (Montagné et al., 2012). SlitO29 demonstrated a rather broad odorant spectrum equivalent to that observed in SSR, with the two best ligands being (E)-ocimene and β-myrcene. Second, comparison of the dose–response curves obtained with the two techniques showed very similar sensitivities: both dose–response curves had similar shapes and slopes, although the responses to (E)-ocimene were observed at a lower threshold in SSR. We observed a slight shift of the dose response curve in favor of the TCI for the pheromonal OR and in favor of the SSR for the generalist OR. However, measures like EC_50_ or Hill coefficient were statistically indistinguishable between the two techniques. These results suggest that TCI can be used for the functional study of specific or general odorant receptors, regardless of the type of *Drosophila* “empty neuron system” used.

The first apparent advantage of imaging is the non-invasive strategy employed to observe neuronal activity. Due to the thinness of the *Drosophila melanogaster* antenna cuticle, it is easy to capture the fluorescence emission from OSNs expressing the calcium indicator GCaMP6 without any dissection, in contrast to electrophysiological approaches which require intrusion with a recording electrode. SSR also involves finding the correct sensillum, which is not always straightforward. Whether the target is at1 or ab3, the selection of the right sensillum is based on visual criteria such as the morphology and location of the sensilla on the antenna but also on the amplitudes and pattern of the action potentials. It is therefore a trial-and-error approach. This process has been partly improved by a GFP fluorescence-guided SSR, which consists in recognizing the correct sensilla through fluorescently targeted olfactory neurons (Lin and Potter, 2015). This crucial step is not necessary with the TCI method. With the UAS/GAL4 system and tissue-specific expression of the calcium indicator, recordings are unambiguously obtained from the correct sensillum type and neurons. In addition, the entire population of neurons on the surface of one side of the fly antenna is recorded, which already offers an averaging step between individual neurons and accordingly lower response variability. Note that we observed some calcium responses in control lines expressing the calcium probe in ab3 sensilla neurons but no OR (except for Orco). Such responses were already observed using SSR in a previous study (Chahda et al., 2019). The origin of these responses is unclear at the moment, but they may be attributed to the presence of ionotropic receptors, such as IR25a, at the membrane of ab3A neurons (Abuin et al., 2011; Grosjean et al., 2011; Rytz et al., 2013; Wicher and Miazzi, 2021; Task et al., 2022). In the absence of a functional OR-Orco complex (as observed in the ab3 control line), it is possible that an IR complex would induce neuronal responses.

In terms of experimental throughput, the time required to prepare the fly for recording is decreased compared to SSR, as it only needs to be placed under the camera (~2 min vs. ~10 min). However, the calcium signals are slow signals that last long after odorant presentation. In the present study, to avoid any influence of one stimulation on the next, we set the inter-trial interval to 2 min, allowing the neurons to return to their basal intracellular Ca^2+^ concentration. In addition, two different calcium probes were tested in this study, GCaMP6s (s for slow) and GCaMP6m (m for medium). The former demonstrated a slower activation rate, increasing the size of the inter-trial interval. This probe also increased the amplitude of the response, which is an advantage for the screening test. The second probe, GCaMP6m, is intermediate and allows a faster activation rate, thus reducing the inter-trial interval. We did not test the third type of probe (GCaMP6f, f for fast) which has the fastest activation rate and may therefore be of interest for reducing the inter-trial interval.

In contrast, in electrophysiology, the time required for neurons to return to their spontaneous electrical activity is usually shorter and recordings are typically performed every 10–30 s (Dobritsa et al., 2003; Kurtovic et al., 2007; Montagné et al., 2012; de Fouchier et al., 2015, 2017; Chahda et al., 2019). Thus, the temporal resolution of the response as well as stimulation frequency are better with SSR. However, we show that imaging allows for the simultaneous placement of several individual flies in the air stream and under the microscope objective. Here, we have shown simultaneous recordings of 2 individuals, but we have already succeeded in making recordings with 4 individuals (data not shown).

The use of SSR coupled to heterologous expression within the *Drosophila* “empty neuron system” has been described as a suitable method for OR deorphanization, and the preparation can last more than 1 h and thus allow large-scale screening compared to other methods such as two electrode voltage-clamp on *Xenopus* oocytes. Nevertheless, the main advantage of the TCI method is the longevity of the fly preparation which is higher than for the SSR method. Here, we subjected individuals to as many as 250 stimulations and about 8 h of recordings. The amplitude of the responses decreased with repeated stimulations, which is partly explained by sensory adaptation (Köster and de Wijk, 1991; Dalton, 2000), but the responses were still above 50% of the initial response after 127 stimulations (about 2 h of recording). Under normal screening conditions, only a few odorants will trigger a response from neurons, so sensory adaptation is expected to be kept to a minimum. Therefore, we expect that the preparation can be maintained for even longer durations than those observed here with repeated presentations of the same stimulus.

In summary, we have expanded the methods available for the functional study of ORs expressed in *Drosophila* olfactory neurons. We have shown that transcuticular calcium imaging can record the same response spectra and dose–response curves with the same sensitivity as SSR. Although based on slow signals, this technique has clear advantages in terms of preparation time and the number of sensilla/animals that can be recorded simultaneously. These advantages could make this technique a suitable first step for large-scale screening of odorants or for the systematic investigation of odorant-detection principles, such as OR co-expression or odor-mixture effects, possibly followed by the use of SSR for a more refined evaluation of OR responses.

## Data availability statement

The raw data are available on request from julia.mariette@universite-paris-saclay.fr.

## Author contributions

TL, NM, TC, and J-CS conceived the experiments. AN and TL collected preliminary data and developed analysis tools. JM and AN collected the data. JM and J-CS analyzed the data and interpreted the results. JM, AN, and J-CS wrote the manuscript. All authors read and edited the manuscript and approved its final version.

## Funding

The study was supported by the ANR (project ANR-17-CE20-003 to J-CS). JM received a PhD grant from the French Research Ministry and additional funding from the Fondation pour la Recherche Médicale (FDT202012020727).

## Conflict of interest

The authors declare that the research was conducted in the absence of any commercial or financial relationships that could be construed as a potential conflict of interest.

## Publisher’s note

All claims expressed in this article are solely those of the authors and do not necessarily represent those of their affiliated organizations, or those of the publisher, the editors and the reviewers. Any product that may be evaluated in this article, or claim that may be made by its manufacturer, is not guaranteed or endorsed by the publisher.

## References

[ref1] Abuin L. Bargeton B. Ulbrich M. H. Isacoff E. Y. Kellenberger S. Benton R. (2011). Functional architecture of olfactory ionotropic glutamate receptors. Neuron 69, 44–60. doi: 10.1016/j.neuron.2010.11.042, PMID: 21220098 PMC3050028

[ref2] Anderson A. R. Wanner K. W. Trowell S. C. Warr C. G. Jaquin-Joly E. Zagatti P.. (2009). Molecular basis of female-specific odorant responses in *Bombyx Mori*. Insect Biochem. Mol. Biol. 39, 189–197. doi: 10.1016/j.ibmb.2008.11.002, PMID: 19100833

[ref3] Andersson M. N. LÃ¶fstedt C. Newcomb R. D. (2015). Insect olfaction and the evolution of receptor tuning. Front. Ecol. Evol. 3:53. doi: 10.3389/fevo.2015.00053

[ref4] Auer T. O. Alvarez-Ocana R. Cruchet S. Benton R. Arguello J. R. (2022). Copy number changes in co-expressed odorant receptor genes enable selection for sensory differences in *Drosophilid* species. Nat. Ecol. Evol. 6:1343-+. doi: 10.1038/s41559-022-01830-y, PMID: 35864227

[ref5] Bavan S. Sherman B. Luetje C. W. Abaffy T. (2014). Discovery of novel ligands for mouse olfactory receptor Mor42-3 using an in silico screening approach and *in vitro* validation. PLoS One 9:E92064. doi: 10.1371/journal.pone.0092064, PMID: 24637889 PMC3956865

[ref6] Benton R. Dahanukar A. (2011). Electrophysiological recording from *Drosophila* olfactory sensilla. Cold Spring Harb. Protoc. 2011, 824–838. doi: 10.1101/pdb.prot5630, PMID: 21724819

[ref7] Brand P. Hinojosa-Diaz I. A. Ayala R. Daigle M. Yurrita Obiols C. L. Eltz T.. (2020). The evolution of sexual signaling is linked to odorant receptor tuning in perfume-collecting orchid bees. Nat. Commun. 11:244. doi: 10.1038/s41467-019-14162-6, PMID: 31932598 PMC6957680

[ref8] Cassau S. Krieger J. (2021). The role of SNMPs in insect olfaction. Cell Tissue Res. 383, 21–33. doi: 10.1007/s00441-020-03336-0, PMID: 33245414 PMC7873011

[ref9] Chahda J. S. Soni N. Sun J. S. Ebrahim S. A. M. Weiss B. L. Carlson J. R. (2019). The molecular and cellular basis of olfactory response to tsetse Fly attractants. PLoS Genet. 15:E1008005. doi: 10.1371/journal.pgen.1008005, PMID: 30875383 PMC6420007

[ref10] Chang H. Unni A. Tom M. T. Llorca L. C. Brase S. Bucks S.. (2022). Non-redundant odorant detection in a locust. bioRxiv. doi: 10.1101/2022.06.21.496967 38070506

[ref11] Claudianos C. Lim J. Young M. Yan S. Cristino A. S. Newcomb R. D.. (2014). Odor memories regulate olfactory receptor expression in the sensory periphery. Eur. J. Neurosci. 39, 1642–1654. doi: 10.1111/ejn.12539, PMID: 24628891

[ref12] Corcoran J. A. Jordan M. D. Carraher C. Newcomb R. D. (2014). A novel method to study insect olfactory receptor function using HEK293 cells. Insect Biochem. Mol. Biol. 54, 22–32. doi: 10.1016/j.ibmb.2014.08.005, PMID: 25174788

[ref13] Couto A. Alenius M. Dickson B. J. (2005). Molecular, anatomical, and functional organization of the *Drosophila* olfactory system. Curr. Biol. 15, 1535–1547. doi: 10.1016/j.cub.2005.07.034, PMID: 16139208

[ref14] Dalton P. (2000). Psychophysical and behavioral characteristics of olfactory adaptation. Chem. Senses 25, 487–492. doi: 10.1093/chemse/25.4.487, PMID: 10944515

[ref15] De Bruyne M. Baker T. C. (2008). Odor detection in insects: volatile codes. J. Chem. Ecol. 34, 882–897. doi: 10.1007/s10886-008-9485-4 18535862

[ref16] De Bruyne M. Foster K. Carlson J. R. (2001). Odor coding in the *Drosophila* antenna. Neuron 30, 537–552. doi: 10.1016/S0896-6273(01)00289-6 11395013

[ref17] De Fouchier A. Sun X. Monsempes C. Mirabeau O. Jacquin-Joly E. Montagné N. (2015). Evolution of two receptors detecting the same pheromone compound in crop pest moths of the genus *Spodoptera*. Front. Ecol. Evol. 3:95. doi: 10.3389/fevo.2015.00095

[ref18] De Fouchier A. Walker Iii W. B. Montagné N. Steiner C. Binyameen M. Schlyter F.. (2017). Functional evolution of Lepidoptera olfactory receptors revealed by deorphanization of a moth repertoire. Nat. Commun. 8:15709. doi: 10.1038/ncomms15709, PMID: 28580965 PMC5465368

[ref19] Dobritsa A. A. Der Goes V. Van Naters W. Warr C. G. Steinbrecht R. A. Carlson J. R. (2003). Integrating the molecular and cellular basis of odor coding in the *Drosophila* antenna. Neuron 37, 827–841. doi: 10.1016/S0896-6273(03)00094-1, PMID: 12628173

[ref20] Ebrahim S. A. M. Dweck H. K. M. Stökl J. Hofferberth J. E. Trona F. Weniger K.. (2015). Drosophila avoids parasitoids by sensing their semiochemicals via a dedicated olfactory circuit. PLoS Biol. 13:E1002318. doi: 10.1371/journal.pbio.1002318, PMID: 26674493 PMC4687525

[ref21] Funakoshi K. Suzuki H. Takeuchi S. J. A. C. (2006). Lipid bilayer formation by contacting monolayers in a microfluidic device for membrane protein analysis. Anal. Chem. 78, 8169–8174. doi: 10.1021/ac0613479 17165804

[ref1001] Gonzalez-Kristeller D. C. Do Nascimento J. B. P. Galante P. A. F. Malnic B. (2015). Identification of agonists for a group of human odorant receptors. Front. Pharmacol. 6. 10.3389/fphar.2015.00035 PMC4347425 25784876

[ref22] Grosjean Y. Rytz R. Farine J. P. Abuin L. Cortot J. Jefferis G.. (2011). An olfactory receptor for food-derived odours promotes male courtship in *Drosophila*. Nature 478, 236–240. doi: 10.1038/nature10428, PMID: 21964331

[ref23] Grosse-Wilde E. Svatos A. Krieger J. (2006). A pheromone-binding protein mediates the bombykol-induced activation of a pheromone receptor *in vitro*. Chem. Senses 31, 547–555. doi: 10.1093/chemse/bjj059, PMID: 16679489

[ref24] Hallem E. A. Carlson J. R. (2006). Coding of odors by a receptor repertoire. Cells 125, 143–160. doi: 10.1016/j.cell.2006.01.050 16615896

[ref25] Hallem E. A. Ho M. G. Carlson J. R. (2004). The molecular basis of odor coding in the *Drosophila* antenna. Cells 117, 965–979. doi: 10.1016/j.cell.2004.05.012, PMID: 15210116

[ref26] Hamana H. Shou-Xin L. Breuils L. Hirono J. Sato T. (2010). Heterologous functional expression system for odorant receptors. J. Neurosci. Methods 185, 213–220. doi: 10.1016/j.jneumeth.2009.09.024 19799933

[ref27] Hansson B. S. (1999). Insect olfaction. Berlin, Germany: Springer Science & Business Media.

[ref28] Hansson B. Stensmyr M. (2011). Evolution of insect olfaction. Neuron 72, 698–711. doi: 10.1016/j.neuron.2011.11.003 22153368

[ref29] Herre M. Goldman O. V. Lu T.-C. Caballero-Vidal G. Qi Y. Gilbert Z. N.. (2022). Non-canonical odor coding in the mosquito. Cells 185, 3104–3123.E28. doi: 10.1016/j.cell.2022.07.024 PMC9480278 35985288

[ref30] Hou X. Zhang D.-D. Yuvaraj J. K. Corcoran J. A. Andersson M. N. Löfstedt C. (2020). Functional characterization of odorant receptors from the moth eriocrania semipurpurella: a comparison of results in the *Xenopus* oocyte and HEK cell systems. Insect Biochem. Mol. Biol. 117:103289. doi: 10.1016/j.ibmb.2019.103289, PMID: 31778795

[ref31] Ignell R. Anton S. Hansson B. S. (2001). The antennal lobe of orthoptera – anatomy and evolution. Brain Behav. Evol. 57, 1–17. doi: 10.1159/000047222, PMID: 11359044

[ref32] Joseph R. M. Carlson J. R. (2015). Drosophila chemoreceptors: a molecular interface between the chemical world and the brain. Trends Genet. 31, 683–695. doi: 10.1016/j.tig.2015.09.005, PMID: 26477743 PMC4674303

[ref1002] Kaiser L. Graveland-Bikker J. Steuerwald D. Vanberghem M. Herlihy K. Zhang S. (2008). Efficient cell-free production of olfactory receptors: Detergent optimization, structure, and ligand binding analyses. PNAS 105, 15726–15731. 18840687 10.1073/pnas.0804766105 PMC2572932

[ref33] Kamikouchi A. Wiek R. Effertz T. Göpfert M. C. Fiala A. (2010). Transcuticular optical imaging of stimulus-evoked neural activities in the *Drosophila* peripheral nervous system. Nat. Protoc. 5, 1229–1235. doi: 10.1038/nprot.2010.85, PMID: 20595952

[ref34] Karner T. Kellner I. Schultze A. Breer H. Krieger J. (2015). Co-expression of six tightly clustered odorant receptor genes in the antenna of the malaria mosquito *Anopheles Gambiae*. Front. Ecol. Evol. 3:36. doi: 10.3389/fevo.2015.00026

[ref35] Katada S. Nakagawa T. Kataoka H. Touhara K. (2003). Odorant response assays for a heterologously expressed olfactory receptor. Biochem. Biophys. Res. Commun. 305, 964–969. doi: 10.1016/S0006-291X(03)00863-5 12767924

[ref36] Kawano R. Osaki T. Sasaki H. Takinoue M. Yoshizawa S. Takeuchi S. J. J. O. T. A. C. S. (2011). Rapid detection of a cocaine-binding aptamer using biological nanopores on a chip. J. Am. Chem. Soc. 133, 8474–8477. doi: 10.1021/ja2026085 21553872

[ref37] Keller A. Vosshall L. B. (2016). Olfactory perception of chemically diverse molecules. BMC Neurosci. 17, 1–17. doi: 10.1186/s12868-016-0287-2 27502425 PMC4977894

[ref38] Khadka R. Aydemir N. Carraher C. Hamiaux C. Colbert D. Cheema J.. (2019). An ultrasensitive electrochemical impedance-based biosensor using insect odorant receptors to detect odorants. Biosens. Bioelectron. 126, 207–213. doi: 10.1016/j.bios.2018.10.043, PMID: 30415156

[ref39] Köster E. De Wijk R. A. (1991). “Olfactory adaptation” in The human sense of smell. eds. Laing D. G. Doty R. L. Breipohl W. (Berlin: Springer), 199–215.

[ref40] Krautwurst D. (2008). Human olfactory receptor families and their odorants. Chem. Biodiv. 5, 842–852. doi: 10.1002/cbdv.200890099 18618407

[ref41] Krautwurst D. Yau K.-W. Reed R. R. (1998). Identification of ligands for olfactory receptors by functional expression of a receptor library. Cells 95, 917–926. doi: 10.1016/S0092-8674(00)81716-X, PMID: 9875846

[ref42] Kruse S. W. Zhao R. Smith D. P. Jones D. N. M. (2003). Structure of a specific alcohol-binding site defined by the odorant binding protein lush from *Drosophila Melanogaster*. Nat. Struct. Biol. 10, 694–700. doi: 10.1038/nsb960, PMID: 12881720 PMC4397894

[ref43] Kurtovic A. Widmer A. Dickson B. J. (2007). A single class of olfactory neurons mediates behavioural responses to a *Drosophila* sex pheromone. Nature 446, 542–546. doi: 10.1038/nature05672, PMID: 17392786

[ref44] Larsson M. C. Domingos A. I. Jones W. D. Chiappe M. E. Amrein H. Vosshall L. B. (2004). Or83b encodes a broadly expressed odorant receptor essential for *Drosophila* olfaction. Neuron 43, 703–714. doi: 10.1016/j.neuron.2004.08.019, PMID: 15339651

[ref1003] Levasseur G. Baly C. Grébert D. Durieux D. Salesse R. Caillol M. (2004). Anatomical and functional evidence for a role of arginine-vasopressin (AVP) in rat olfactory epithelium cells. Eur. J. Neurosci. 20, 658–670. 15255977 10.1111/j.1460-9568.2004.03516.x

[ref45] Levasseur G. Persuy M.-A. Grebert D. Remy J.-J. Salesse R. Pajot-Augy E. (2003). Ligand-specific dose–response of heterologously expressed olfactory receptors. Eur. J. Biochem. 270, 2905–2912. doi: 10.1046/j.1432-1033.2003.03672.x 12823561

[ref46] Li Q. Liberles S. D. (2015). Aversion and attraction through olfaction. Curr. Biol. 25, R120–R129. doi: 10.1016/j.cub.2014.11.044 25649823 PMC4317791

[ref47] Lin C.-C. Potter C. J. (2015). Re-classification of *Drosophila melanogaster* trichoid and intermediate sensilla using fluorescence-guided single sensillum recording. PLoS One 10:E0139675. doi: 10.1371/journal.pone.0139675, PMID: 26431203 PMC4592000

[ref48] Liu Y. Chen Q. Man Y. H. Wu W. J. (2013). Insect olfactory receptors as essential detectors for volatile chemicals in biomimetic odorant sensors. Appl. Mech. Mater. 461, 822–828. doi: 10.4028/www.scientific.net/AMM.461.822

[ref49] Lundin C. Käll L. Kreher S. A. Kapp K. Sonnhammer E. L. Carlson J. R.. (2007). Membrane topology of the *Drosophila* Or83b odorant receptor. FEBS Lett. 581, 5601–5604. doi: 10.1016/j.febslet.2007.11.007, PMID: 18005664 PMC2176074

[ref50] Misawa N. Fujii S. Kamiya K. Osaki T. Takaku T. Takahashi Y.. (2019). Construction of a biohybrid odorant sensor using biological olfactory receptors embedded into bilayer lipid membrane on a chip. ACS Sens 4, 711–716. doi: 10.1021/acssensors.8b01615, PMID: 30829476

[ref51] Montagné N. Chertemps T. Brigaud I. Francois A. Francois M. C. De Fouchier A.. (2012). Functional characterization of a sex pheromone receptor in the pest moth *Spodoptera littoralis* by heterologous expression in *Drosophila*. Eur. J. Neurosci. 36, 2588–2596. doi: 10.1111/j.1460-9568.2012.08183.x, PMID: 22748123

[ref52] Murugathas T. Zheng H. Y. Colbert D. Kralicek A. V. Carraher C. Plank N. O. V. (2019). Biosensing with insect odorant receptor nanodiscs and carbon nanotube field-effect transistors. ACS Appl. Mater. Interfaces 11, 9530–9538. doi: 10.1021/acsami.8b19433, PMID: 30740970

[ref53] Osaki T. Takeuchi S. J. A. C. (2017). Artificial cell membrane systems for biosensing applications. Anal. Chem. 89, 216–231. doi: 10.1021/acs.analchem.6b04744 27959515

[ref54] Parker E. M. Grisel D. A. Iben L. G. Nowak H. P. Mahle C. D. Yocca F. D.. (1994). Characterization of human 5-HT1 receptors expressed in Sf9 insect cells. Eur. J. Pharmacol. Mol. Pharmacol. 268, 43–53. doi: 10.1016/0922-4106(94)90118-X, PMID: 7925611

[ref55] Pelz D. Roeske T. Syed Z. Bruyne M. D. Galizia C. G. (2006). The molecular receptive range of an olfactory receptor *in vivo* (*Drosophila Melanogaster* Or22a). J. Neurobiol. 66, 1544–1563. doi: 10.1002/neu.20333 17103386

[ref56] Peterlin Z. Firestein S. Rogers M. E. (2014). The state of the art of odorant receptor deorphanization: a report from the orphanage. J. Gen. Physiol. 143, 527–542. doi: 10.1085/jgp.201311151, PMID: 24733839 PMC4003190

[ref57] Pitts S. Pelser E. Meeks J. Smith D. (2016). Odorant responses and courtship behaviors influenced by at4 neurons in *Drosophila*. PLoS One 11:E0162761. doi: 10.1371/journal.pone.0162761, PMID: 27617442 PMC5019410

[ref58] Prelic S. Pal Mahadevan V. Venkateswaran V. Lavista-Llanos S. Hansson B. S. Wicher D. (2022). Functional interaction between *Drosophila* olfactory sensory neurons and their support cells. Front. Cell. Neurosci. 15:789086. doi: 10.3389/fncel.2021.789086, PMID: 35069116 PMC8777253

[ref59] Ritz S. Hulko M. Zerfaß C. May S. Hospach I. Krasteva N.. (2013). Cell-free expression of a mammalian olfactory receptor and unidirectional insertion into small unilamellar vesicles (SUVs). Biochimie 95, 1909–1916. doi: 10.1016/j.biochi.2013.06.021, PMID: 23816872

[ref60] Robertson H. M. Robertson E. C. N. Walden K. K. O. Enders L. S. Miller N. J. (2019). The chemoreceptors and odorant binding proteins of the soybean and pea aphids. Insect Biochem. Mol. Biol. 105, 69–78. doi: 10.1016/j.ibmb.2019.01.005 30654011

[ref61] Rolls E. T. Tovee M. J. (1995). Sparseness of the neuronal representation of stimuli in the primate temporal visual cortex. J. Neurophysiol. 73, 713–726. doi: 10.1152/jn.1995.73.2.713, PMID: 7760130

[ref62] Rytz R. Croset V. Benton R. (2013). Ionotropic receptors (IRS): chemosensory ionotropic glutamate receptors in *Drosophila* and beyond. Insect Biochem. Mol. Biol. 43, 888–897. doi: 10.1016/j.ibmb.2013.02.007 23459169

[ref63] Sakurai T. Mitsuno H. Haupt S. S. Uchino K. Yokohari F. Nishioka T.. (2011). A single sex pheromone receptor determines chemical response specificity of sexual behavior in the silkmoth *Bombyx mori*. PLoS Genet. 7:E1002115. doi: 10.1371/journal.pgen.1002115 21738481 PMC3128102

[ref64] Sato K. Pellegrino M. Nakagawa T. Nakagawa T. Vosshall L. B. Touhara K. (2008). Insect olfactory receptors are heteromeric ligand-gated ion channels. Nature 452, 1002–1006. doi: 10.1038/nature06850, PMID: 18408712

[ref65] Shirokova E. Schmiedeberg K. Bedner P. Niessen H. Willecke K. Raguse J. D.. (2005). Identification of specific ligands for orphan olfactory receptors. G protein-dependent Agonism and antagonism of odorants. J. Biol. Chem. 280, 11807–11815. doi: 10.1074/jbc.M411508200, PMID: 15598656

[ref66] Steinbrecht R. A. (1992). Experimental morphology of insect olfaction: tracer studies, X-ray microanalysis, autoradiography, and immunocytochemistry with silkmoth antennae. Microsc. Res. Tech. 22, 336–350. doi: 10.1002/jemt.1070220404, PMID: 1392064

[ref67] Strauch M. Lüdke A. Münch D. Laudes T. Galizia C. G. Martinelli E.. (2014). More than apples and oranges - detecting cancer with a fruit fly's antenna. Sci. Rep. 4:3576. doi: 10.1038/srep03576, PMID: 24389870 PMC3880960

[ref68] Task D. Lin C. C. Vulpe A. Afify A. Ballou S. Brbic M.. (2022). Chemoreceptor co-expression in *Drosophila melanogaster* olfactory neurons. Elife 11:e72599. doi: 10.7554/eLife.72599, PMID: 35442190 PMC9020824

[ref69] Tegler L. T. Corin K. Hillger J. Wassie B. Yu Y. Zhang S. (2015). Cell-free expression, purification, and ligand-binding analysis of *Drosophila Melanogaster* olfactory receptors DmOR67a, DmOR85b and DmORCO. Sci. Rep. 5:7867. doi: 10.1038/srep07867, PMID: 25597985 PMC4297953

[ref70] Thach T. T. Hong Y.-J. Lee S. Lee S.-J. (2017). Molecular determinants of the olfactory receptor Olfr544 activation by azelaic acid. Biochem. Biophys. Res. Commun. 485, 241–248. doi: 10.1016/j.bbrc.2017.02.104, PMID: 28235481

[ref71] Thurm U. Küppers J. (1980). “Epithelial physiology of insect Sensilla” in Insect biology in the future. (Cambridge, Massachusetts, United States: Academic Press), 735–763.

[ref72] Tsitoura P. Andronopoulou E. Tsikou D. Agalou A. Papakonstantinou M. P. Kotzia G. A.. (2010). Expression and membrane topology of *Anopheles Gambiae* odorant receptors in lepidopteran insect cells. PLoS One 5:E15428. doi: 10.1371/journal.pone.0015428, PMID: 21082026 PMC2972716

[ref73] Vosshall L. B. Hansson B. S. (2011). A unified nomenclature system for the insect olfactory coreceptor. Chem. Senses 36, 497–498. doi: 10.1093/chemse/bjr022, PMID: 21441366

[ref74] Wang B. Liu Y. He K. Wang G. (2016). Comparison of research methods for functional characterization of insect olfactory receptors. Sci. Rep. 6:32806. doi: 10.1038/srep32806, PMID: 27633402 PMC5025650

[ref75] Wanner K. W. Nichols A. S. Allen J. E. Bunger P. L. Garczynski S. F. Linn C. E.. (2010). Sex pheromone receptor specificity in the European corn borer moth, *Ostrinia nubilalis*. Plos One 5:e8685. doi: 10.1371/journal.pone.0008685 20084285 PMC2801615

[ref76] Wanner K. W. Nichols A. S. Walden K. K. O. Brockmann A. Luetje C. W. Robertson H. M. (2007). A honey bee odorant receptor for the queen substance 9-oxo-2-decenoic acid. Proc. Natl. Acad. Sci. U. S. A. 104, 14383–14388. doi: 10.1073/pnas.0705459104, PMID: 17761794 PMC1964862

[ref77] Wetzel C. H. Oles M. Wellerdieck C. Kuczkowiak M. Gisselmann G. Hatt H. (1999). Specificity and sensitivity of a human olfactory receptor functionally expressed in human embryonic kidney 293 cells and *Xenopus laevis* oocytes. J. Neurosci. 19, 7426–7433. doi: 10.1523/JNEUROSCI.19-17-07426.1999, PMID: 10460249 PMC6782526

[ref78] Wicher D. Miazzi F. (2021). Functional properties of insect olfactory receptors: ionotropic receptors and odorant receptors. Cell Tissue Res. 383, 7–19. doi: 10.1007/s00441-020-03363-x, PMID: 33502604 PMC7873100

[ref79] Wicher D. Schäfer R. Bauernfeind R. Stensmyr M. C. Heller R. Heinemann S. H.. (2008). Drosophila odorant receptors are both ligand-gated and cyclic-nucleotide-activated cation channels. Nature 452, 1007–1011. doi: 10.1038/nature06861, PMID: 18408711

[ref80] Xu P. Atkinson R. Jones D. N. M. Smith D. P. (2005). Drosophila OBP LUSH is required for activity of pheromone-sensitive neurons. Neuron 45, 193–200. doi: 10.1016/j.neuron.2004.12.031, PMID: 15664171

[ref81] Xu W. Papanicolaou A. Liu N. Y. Dong S. L. Anderson A. (2015). Chemosensory receptor genes in the oriental tobacco budworm *Helicoverpa assulta*. Insect Mol. Biol. 24, 253–263. doi: 10.1111/imb.12153, PMID: 25430896

[ref82] Yan H. Jafari S. Pask G. Zhou X. Reinberg D. Desplan C. (2020). Evolution, developmental expression and function of odorant receptors in insects. J. Exp. Biol. 223:jeb208215. doi: 10.1242/jeb.208215 32034042 PMC7790194

[ref83] Yoshii T. Takayama I. Fukutani Y. Ikuta T. Maehashi K. Yohda M. (2022). Development of an odorant sensor with a cell-free synthesized olfactory receptor and a graphene field-effect transistor. Anal. Sci. 38, 241–245. doi: 10.1007/s44211-022-00073-y 35286654

[ref84] Zhao H. Ivic L. Otaki J. M. Hashimoto M. Mikoshiba K. Firestein S. (1998). Functional expression of a mammalian odorant receptor. Science 279, 237–242. doi: 10.1126/science.279.5348.237 9422698

