# Supplementary material for: Transcuticular calcium imaging as a tool for the functional study of insect odorant receptors
Source: Front Mol Neurosci. 2023 Aug 14;16:1182361. doi: 10.3389/fnmol.2023.1182361 (PMC10461100; doi:10.3389/fnmol.2023.1182361)
Supplement: Supplementary file 1 [file Data_Sheet_1.PDF]

Supplementary materials

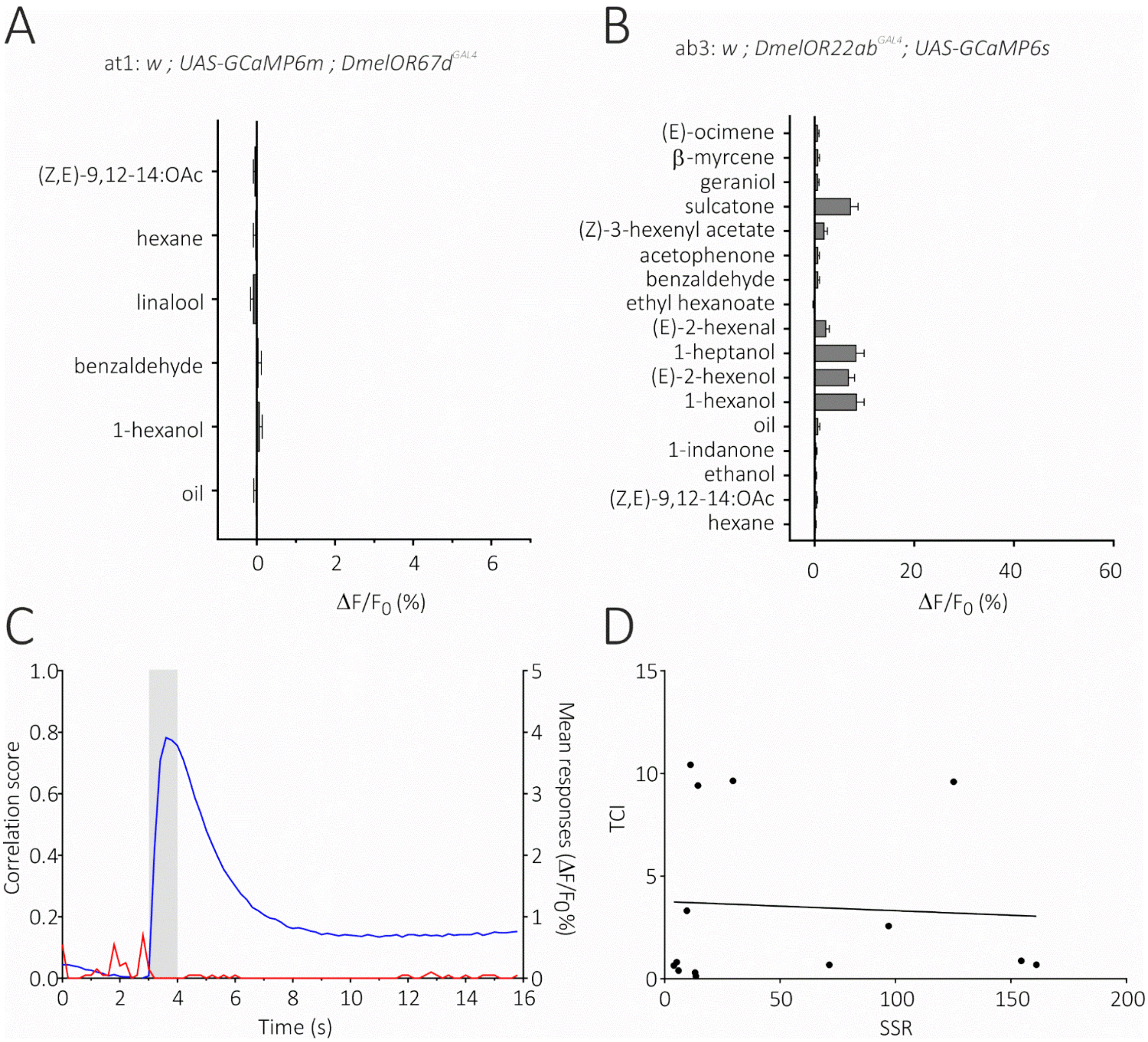

Figure 1: Supplementary materials. Electrophysiological recordings (SSR) from at1 sensilla of UAS-GCaMP6m flies (Friedman test, n = 12, $p < 0.0001$) **(A)** and from ab3 sensilla of UAS-GCaMP6s flies **(B)** which do not express odorant receptors (*empty neuron*) (Friedman test, n = 12, $p > 0.05$). (Friedman test, n = 12, $p < 0.01$). Some responses are significantly higher than to the control (1-heptanol, (E)-2-hexenol, 1-hexanol and sulcatone). (**C**) (**D**) Cross-correlation analysis between SSR data and the genetic control calcium imaging data (red curve, left Y axis). During odor delivery, average response amplitude increased (blue curve, right Y axis), but the correlation coefficient remained very low, showing that the responses of the genetic control line could not explain the high correlation observed with the SlitOR29 expressing line (compare with Fig 6B).
